# Self-Assembly of Mesoscale Isomers: The Role of Pathways and Degrees of Freedom

**DOI:** 10.1371/journal.pone.0108960

**Published:** 2014-10-09

**Authors:** Shivendra Pandey, Daniel Johnson, Ryan Kaplan, Joseph Klobusicky, Govind Menon, David H. Gracias

**Affiliations:** 1 Department of Chemical and Biomolecular Engineering, The Johns Hopkins University, Baltimore, Maryland, United States of America; 2 Division of Applied Mathematics, Brown University, Providence, Rhode Island, United States of America; Brandeis University, United States of America

## Abstract

The spontaneous self-organization of conformational isomers from identical precursors is of fundamental importance in chemistry. Since the precursors are identical, it is the multi-unit interactions, characteristics of the intermediates, and assembly pathways that determine the final conformation. Here, we use geometric path sampling and a mesoscale experimental model to investigate the self-assembly of a model polyhedral system, an octahedron, that forms two isomers. We compute the set of all possible assembly pathways and analyze the degrees of freedom or rigidity of intermediates. Consequently, by manipulating the degrees of freedom of a precursor, we were able to experimentally enrich the formation of one isomer over the other. Our results suggest a new approach to direct pathways in both natural and synthetic self-assembly using simple geometric criteria. We also compare the process of folding and unfolding in this model with a geometric model for cyclohexane, a well-known molecule with chair and boat conformations.

## Introduction

Structural isomers are an important class of molecules with the same chemical formula but varied geometric arrangements of bonds, resulting in different physical and chemical properties [1]. For example, n-pentane, isopentane and neopentane all have five carbon atoms, twelve hydrogen atoms and similar interatomic bonding (tetrahedral *sp^3^* carbon). However, due to a molecular positional difference, the three conformations have significantly different boiling and melting points [2]. Likewise, isomerization of a single amino acid such as proline can have a dramatic influence on the assembly of larger ribonuclease and consequently cause slow folding of the molecule [3], [4]. While it has been empirically established that catalysts can be used to enrich one isomer over another [5], [6], the role of geometry, steric interactions in intermediates and assembly pathways are not well understood. Consequently, mechanisms and rational designs to synthetically enrich one self-assembling isomer over the other are limited.

**Figure 1:**
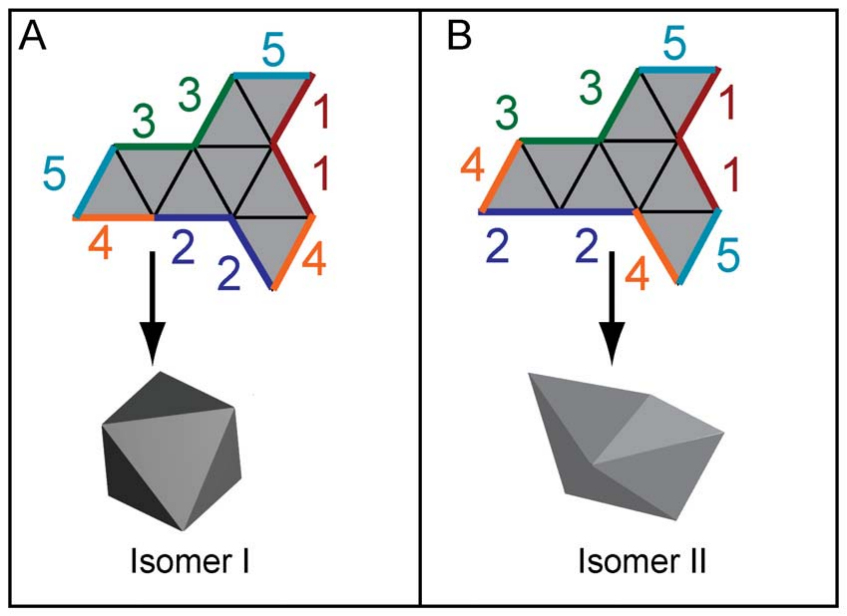
Schematic illustration of the self-assembly of two different octahedral isomers from the same initial precursor or net. (A) Isomer I is a convex octahedron, while (B) Isomer II is a non-convex octahedron. Both isomers can form from the same precursor net as depicted. Individual isomers form via a different assembly sequence when different edge pairs denoted by 1, 2, 3, 4, and 5 meet. In Isomer I, each vertex is bonded to four other vertices through edge connections whereas in Isomer II, two vertices have four edge connections, two vertices have five edge connections and the remaining two vertices have three edge connections.

In this article, we investigate the self-assembly of isomers in a mesoscale model system. Self-assembly is a technique to create higher order complex structures from multiple subunits. These subunits can be biomolecules, nanostructures or microscale and millimeter scale structures [7]–[14]. Here, the mesoscale model involves the self-assembly of 300 µm sized polygonal units into polyhedra using surface tension driven forces that both fold panels and seal edges [15], [16]. We focus on the self-assembly of an octahedron, a Platonic polyhedron which can be constructed by folding a planar assembly of eight triangles termed a net as shown in Fig. 1. Despite its simplicity, we study the octahedron, because its nets can also be folded into a second non-convex conformation, akin to a boat, as illustrated in Fig. 1. These two three-dimensional shapes have the same precursor, but are formed by different bonding arrangements between edges. Thus, they are structural isomers. In what follows, we refer to the (convex) octahedron as Isomer I and the non-convex ‘boat’ as Isomer II. We investigate the formation of these isomers with theory and experiment. Further, we analyze the degrees of freedom of intermediate states and experimentally demonstrate how a precursor can be manipulated to increase the yield of formation of Isomer II.

Mesoscale models of self-assembly [17] provide a valuable tool to investigate pathways and intermediates for unit interactions in nucleic acids [18], molecular folding, and molecule-mimetic chemistry [19]. They provide a means to visualize self-assembly in real time, elucidate the effects of geometry and characterize interactions and intermediates. Our model has several attractive features; (a) precursors are lithographically fabricated with high throughput, versatility and precision. Hence, variations in size, shape and weight can be readily introduced and units can be mass-produced and arranged in any desired planar geometry; (b) self-assembly occurs merely on heating and does not involve any human intervention such as pneumatic or electrical wiring or controls that could bias a particular pathway or sequence; (c) the relative strength of the mechanical agitation can be tuned relative to the strength of the interactions so that some kinetically trapped states can be avoided. Consequently, the model resembles a mechanical analog of the self-assembly of polyhedral molecules in a thermally agitated environment (kT); (d) self-assembly occurs due to a hierarchy of primary and secondary interactions thus providing an analogy to hierarchical interactions such as those involving covalent, electrostatic, steric and van der Waals forces in molecular self-assembly.

The use of solid polyhedra to model molecular conformations may be less familiar today than ball-and-stick models, but such models are deeply rooted in chemistry. In fact, they were first used by Jacobus van't Hoff in the 19th century to suggest that molecules have three dimensional spatial structures. van't Hoff used solid tetrahedra to model *sp^3^* carbon atoms and glued them together to build polyhedral models for larger molecules such as tartaric acid, malic acid, and succinic acid [20]. It is important to note that many salts, organometallic compounds, inorganic clusters and biomolecules exist in polyhedral geometries [21]–[24]. For example, the Platonic and Archimedean solids describe the geometry of several organic molecules such as tetrahedrane [25], cubane [26], octahedrane [27], dodecahedrane [28], fullerene [29], supramolecular cages [30] and icosahedral viruses [31]. Also, in synthetic chemistry, polyhedral geometries of organometallic compounds have been used to study the role of geometric constraints on the self-assembled macromolecules [32]–[35]. In addition, folding patterns also played an important role in early analyses of the conformational isomers of cyclohexane. Hermann Sachse in 1890, folded paper models of ideal chair and boat conformations of cyclohexane to demonstrate the fact that allowing two carbon atoms to lie outside the plane could alleviate the angle strain in cyclohexane molecules [36], [37] (Images of our replication of his paper models are contrasted with ball-and-stick models in Fig. S1). As for polyhedral isomers in nature, we note that Si_6_ exists as both a convex octahedron as well as a non-convex boat [38].

## Results and Discussion

This work consists of two main parts: (a) explicit enumeration and analysis of all intermediates and folding pathways that emerge from octahedral nets; (b) an experimental study of self-assembly from these nets that exploits the degrees of freedom of an intermediate to enrich the formation of one isomer over the other. Finally, we also briefly contrast our results with Sachse's geometric model of cyclohexane.

### 1. The configuration space for the octahedron

An octahedron can form by the folding of 11 planar nets, all of which are investigated in our study. We ask the critical question: What geometric features, pathways and intermediates cause nets to self-assemble into Isomer I or II? In order to investigate the assembly pathways and intermediates that emerge from these nets we modeled the assembly using discrete geometry, extending ideas introduced earlier [16]. In this approximation, we ignore the elastic deformation of each polygonal panel (or face) of the net, treat the internal hinges between panels as ideal hinges that allow free rotation, and the external hinges as ideal sticky edges – that is, two such edges glue perfectly and seal the panels when they meet. We further approximate the continuous process of the folding of a net into a polyhedron by a finite set of discrete, partially formed intermediate states that account for the edges that have been glued. The graph consisting of all states and links between them is called the configuration space, *Є*. The pathways of assembly are modeled as paths in this graph that originate at a net and end in a state that can be folded no further.

The discretization of states relies on the notion of a vertex connection. A vertex on an intermediate state (including a net) is counted as a vertex connection if it is shared by two panels that do not share any edges. The main feature of the octahedral nets that allows the formation of isomers is that there are two different types of vertex connections, one with an angle of 120° between edges and the other with an angle of 180°. For example, in Fig. 1A, vertices shared by edge pairs denoted by 1, 2 and 3 are vertex connections with 120° angles between them. In Fig. 1B, the vertex connection shared by edge pair 2 makes an angle of 180°, whereas the other vertex connections between edge pairs 1 and 3 have angles of 120°. In order to construct the configuration space for octahedral self-assembly, we begin at a net and proceed with an algorithm which involves gluing at vertex connections, as detailed in the supporting information (Text S1). This geometric algorithm captures the essential features of the self-folding process used in our experiments.

**Figure 2:**
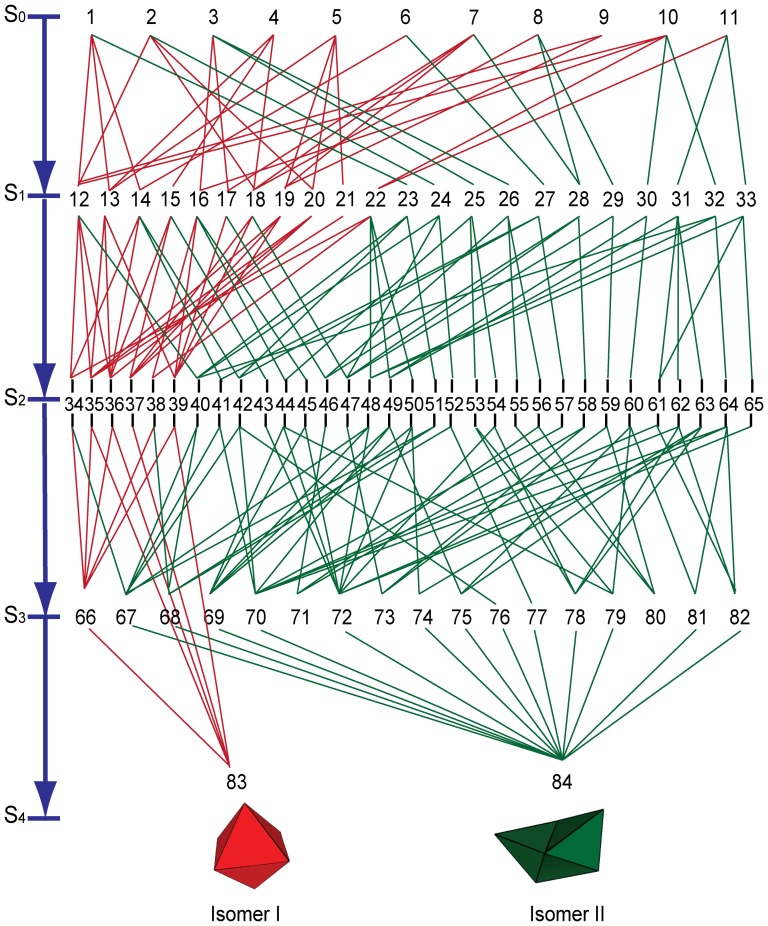
The extended configuration space 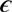 for the octahedron showing intermediates divided into five tiers (denoted by S_0_ to S_4_). The intermediates in tier *S_k_* have *(4-k)* internal degrees of freedom. The paths denoted in red correspond to states linked by gluing at vertex connections with exterior angle 120° (configuration space *R*); all these paths lead to the formation of Isomer I. The paths in green link states obtained by gluing at both types of vertex connections (configuration space *G*). These paths lead to Isomer II, and kinetically trapped states 71, 73 and 80.

We computed the configuration space by gluing at vertex connections with exterior angle of either 120° or 180° until a final folded state is reached. The complete set of states *Є* that results from all 11 nets of the octahedron consists of the 84 states, and links between them are shown in Fig. 2. (Corresponding shapes for each numbered configuration are shown in Fig. S2). The configuration space naturally divides into five tiers (denoted by S_0_ to S_4_). The states in each tier are obtained from the states in the tier above by gluing one pair of edges. Further, *Є* can be divided into two subsets. The subset denoted by *R* (red connections in Fig. 2) results from gluing only vertex connections that subtend angles of 120°, whereas the remaining states denoted by *G* (green connections in Fig. 2) result from gluing vertex connections that subtend angles of both 120° and 180°. Only the red connections lead to Isomer I whereas most green connections lead to Isomer II. In addition, the vertices in the three intermediates 71, 73 and 80 are glued together in such a way that no further assembly is possible, despite the fact that not all edges have been glued. Thus, these are also terminal states that are kinetically trapped, but we find them less relevant in experiment. When comparing with experiment, previously published heuristics were used to focus on a few dominant intermediates (Text S2).

### 2. Degrees of freedom and rigidity within intermediates

It is intuitively clear that a flat octahedron net is flexible, whereas an assembled octahedron is rigid. In particular, the rigidity of the octahedron follows from a classical theorem of Cauchy which states that a convex polyhedron is rigid [39]. However, the first example of a non-convex flexible polyhedron was discovered by Connelly in 1977 [40]. This idea of rigidity can be quantified in the mathematical theory of rigidity of ideal linkages as discussed below. An ideal polyhedral linkage consists of a collection of rigid panels that are connected by ideal hinges that allow free rotation. The *number of degrees of freedom* of a linkage is the difference between the number of coordinates required to specify all its vertices, and the number of constraint equations. A rigid body has six degrees of freedom – three coordinates for its center of mass and three for its orientation. The number of *internal degrees of freedom* is the number of degrees of freedom minus six. In more intuitive terms, the number of internal degree of freedom is the number of independent relative motions of a linkage. By this reckoning, we find that the intermediates in tier *S_k_* have *(4-k)* internal degrees of freedom. Thus, in each step of the assembly process the number of internal degrees of freedom decreases by one, until the assembly process terminates in a rigid state. However, these notions do not distinguish between the intermediates on the same tier. In order to distinguish between these intermediates, we note that rigidity theory may be further applied to sub-linkages within a linkage. More precisely, for any given linkage, we may compute the number of internal degrees of freedom of a subset of the linkage. We focus on a sub-linkage consisting of the faces that meet at the corner (vertex) of a polyhedron. Note that the corners of a tetrahedron, cube and dodecahedron are rigid. For example, the corner of a tetrahedron is a linkage consisting of three triangles meeting at a dihedral angle of 70.53°. It is easy to verify that this linkage cannot be deformed to another shape while keeping the edge length fixed. In contrast, the corner of an octahedron has one rotational degree of freedom. It is this relative motion of the panels or this internal degree of freedom that allows for the formation of octahedral isomers as self-assembly proceeds. All the red pathways in Fig. 2 correspond to the formation of ‘octahedral corners’ obtained by gluing at vertex connections with an angle of 120°. Each of these corners has one internal degree of freedom. However, gluing at vertex connections with an angle of 180° leads to a ‘tetrahedral corner’ which is rigid. Thus, while the red and green states on the tier *S_k_* have the same total number of degrees of freedom *(4-k)*, the relative mobility of their corners is completely different, and the partial rigidity of these states is different. Further quantification of these ideas is discussed in the supporting information (Text S3).

### 3. Self-assembly experiments

**Figure 3:**
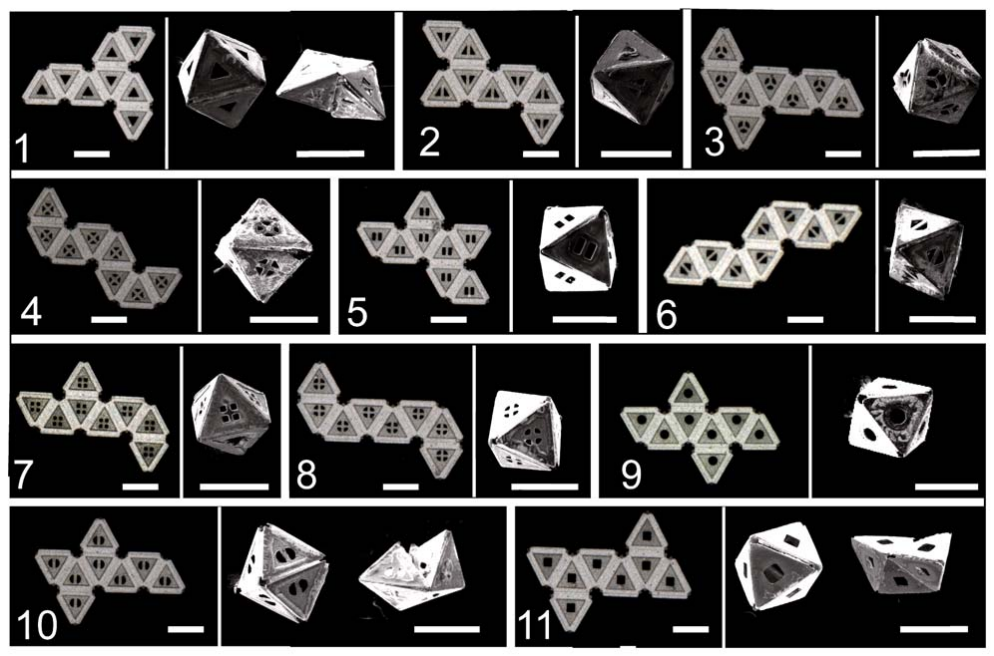
Experimental self-assembly results. Optical (left) and scanning electron microscopy (right) images of all 11 octahedron nets and their self-assembled isomers, I and II. The scale bar is 300 µm.

We investigated the validity of our theoretical approach using experiments on self-assembly of 300 µm sided octahedra following a previously published experimental protocol [13]. As in our theoretical model, we observed that although 11 precursors have the same number of degrees of freedom, only certain precursors formed different isomers. Experimentally, we observed that only the precursors 1, 10 and 11 formed both Isomers I and II (Fig. 3), while precursors 2, 3, 4, 5, 6, 7, 8 and 9 formed only Isomer I. Overall, the yield data suggests a higher propensity to form Isomer I. Snapshots of the self-assembly pathways of an octahedron net (net 10) into Isomer II is shown in Fig. S3. They show the formation of the rigid intermediate 72 on assembly from net 10 to Isomer II, in agreement with the dominant intermediate shown in our models (Fig. S4). Here, it is important to note that several nets formed Isomer II (dihedral angles 31.59°, 109.47° and 218.94°) despite the fact that the amount of hinge material was optimized for formation of Isomer I (dihedral angle of 109.47°) only. In addition, during self-assembly of net 10, we observed that a delay in the rotation of an outer panel gives the other panels an opportunity to move and adjust dihedral angles to form Isomer II (Movie S1).

### 4. Engineering pathways to enrich isomers

**Figure 4:**
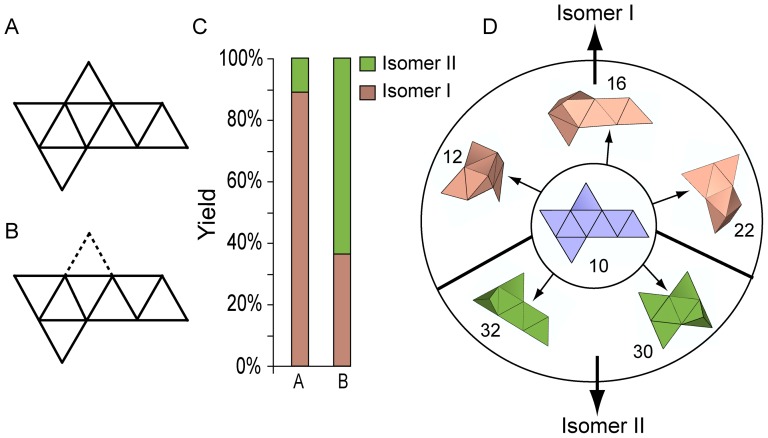
Geometric manipulation of identical precursor nets to manipulate pathways to enrich an isomer. The net 10 shown in (A) and (B) have identical placement of panels but a single panel indicated by the dotted line is excluded to enhance pathways which feature a propensity for 180° folds. (C) Experimentally obtained yields showing a dramatic increase in Isomer II and decrease in Isomer I for the engineered net with a standard deviation of 2.6%. (D) Images of the precursor net 10 and first tier S_1_ intermediates. Pathways which feature intermediates (shown in green color) and form Isomer II are enriched for net B as compared to net A.

Since we observed that the partial rigidity of intermediates and the associated pathways were important in determining which isomer self-assembled, we hypothesized that it might be possible to selectively enrich the formation of one isomer over the other by manipulating these geometric criteria. Importantly, since we observed that Isomer II formed through rigid intermediates formed only via 180° folds, we experimentally compared the relative formation of isomers from two identical nets (net 10, Fig. 4) with just one difference. Essentially, one panel that is important in maintaining rigidity of partially folded modules of intermediates via 120° folds was omitted and the self-assembly yields were compared. In essence by removing this panel, we are eliminating two vertex connections and reducing the degrees of freedom of the partially folded intermediate in the first tier (S_1_). We note that the self-assembly of the net with one panel omitted (Fig. 4B) results into the formation of octahedral isomers I and II with one open face. Consequently, we bias the pathway that occurs during the delay in rotation of the outer panels as noted earlier (Movie S1).

This hypothesis was tested experimentally. After self-assembly of a sample set of 50 for each of the two nets shown in Fig. 4A and B, the polyhedra were carefully examined under an optical microscope and categorized into three grades: grade A with no defects observed under the microscope, grade B with panels misaligned by an angle of 20° or less, and grade C includes polyhedra with multiple defects. Remarkably, the fraction of perfect isomeric polyhedra was dramatically different for the two cases. Even though the geometric placement of the panels in the net is identical, just by removing this one panel, the yield of A grade Isomer I decreased by a factor of two and simultaneously the yield of A grade Isomer II increased by a factor of six (Fig. 4; representative images of octahedral isomers and experimental data are shown in Fig. S5). As depicted in Fig. 4D, on removal of the panel, the pathway with intermediates 30 and 32 is enriched over the pathways with intermediates 12, 16 and 22. This experiment highlights how the degrees of freedom of intermediates and the pathways can be engineered by manipulating the geometric constraints of initial precursors to enrich one isomer over another.

### 5. Analogy between deformation of octahedral isomers and chair/boat transition of cyclohexane

Some of the earliest studies directed at explaining the formation of the two isomers of cyclohexane used paper origami [40]. In order to relate our work to this fundamental example in stereochemistry, we revisit the conformational analysis of cyclohexane from the point of view of the theory of linkages. We compare two ideal linkages: a polyhedral linkage that can be folded into Isomers I and II as in our model experimental system, and an ideal geometric model of cyclohexane.

**Figure 5:**
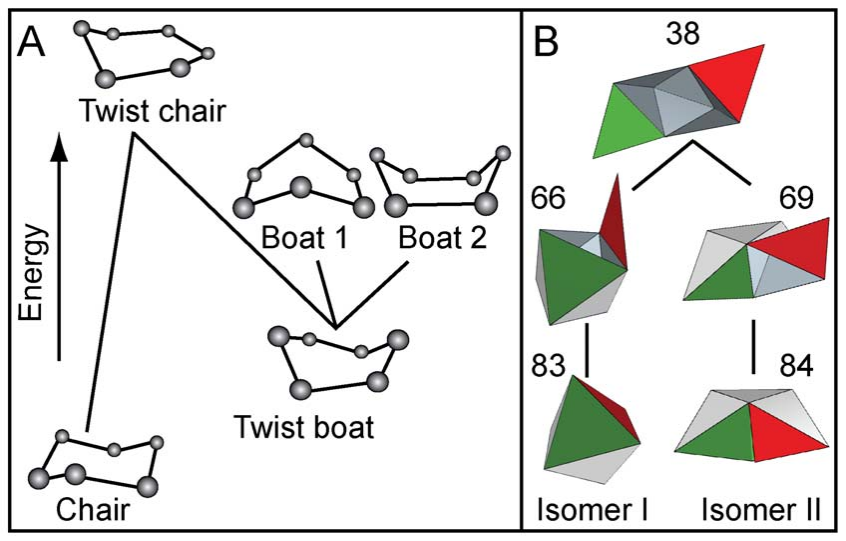
Analogy between (A) the pathways for transition between chair and boat isomers of cyclohexane, and (B) an example of the pathways for transition between octahedral isomers I and II.

Cyclohexane (C_6_H_12_) is a molecule composed of six carbon and twelve hydrogen atoms. The carbon atoms are connected in a ring with two hydrogen atoms attached to each carbon atom. Each carbon has four bonds that energetically prefer spacing at tetrahedral angles. While the actual configurations of cyclohexane balance various effects such as eclipsing strain, angle and steric crowding, we use Sachse's ideal geometric model to facilitate a comparison with polyhedral linkages. We consider the carbon atoms linked by bonds of a fixed length that are required to meet at a fixed angle and we ask which configurations can be deformed into others while preserving these constraints. We find that the chair has zero degrees of freedom whereas the boat has one (Text S4). This means that it is impossible to transform the chair into any other configuration without deforming the bond lengths or angles. Note that this is a purely kinematic argument for the greater stability of the chair form of cyclohexane. However, since the boat has one degree of freedom, it can be deformed continuously while keeping the bond lengths and bond angles fixed. Three such intermediate configurations are shown in Fig. 5A (boat 1, the twist boat and the twist chair). In Sachse's ideal geometric model, the chair is rigid; therefore it cannot transition into the boat. In reality, thermal effects allow fluctuations in bond lengths. To transition between the chair and boat configuration, the chair model must first become a twist chair and then a twist boat before it can finally become boat 1 or boat 2, which differ only by which of the six carbon atoms form the tips of the boats. Similarly, to transition between two boats, the twist boat intermediate must be visited.

For the analogous transformation of the octahedron between Isomer I and Isomer II, it is necessary for an edge to break (unfold), the linkage to move, and the edges to refold. This can occur along many pathways (Fig. 2) and one such example is shown in Fig. 5B. At present, the gluing of hinges in our model experiment is irreversible. Nevertheless, the analogy between these two polyhedral systems suggests that it is possible for octahedral isomers to transition from one to another by choosing appropriate (flexible) hinge materials and mechanical agitation.

## Conclusions

A mesoscale experimental model and the theory of polyhedral linkages have been used to study the self-assembly of structural isomers, and to design an experiment that demonstrates the preferential enrichment of one isomer over the other. We report an important finding of relevance to self-assembly systems: the pathways that proceed through intermediates with favorable rigidity dominate the self-assembly process. We show how isomer enrichment can be achieved by manipulation of the degrees of freedom of initial precursors using purely geometric criterion to bias specific assembly pathways. We achieved this by the removal of a panel and consequently two 120° vertex connections so that the rotation sequence of panels could be controlled to follow pathways leading to Isomer II. Steric and geometric manipulations of molecules are known to be important in molecular catalysis and these ideas are in agreement with our findings. Our findings also suggest that the design of systems that alter the degrees of freedom of precursors and intermediates are important in synthetic self-assembly. The development of approaches to search for and design rigid or stable intermediates could prove useful in solving many inverse problems in self-assembly. Consequently, steric hindrances or more rigid geometric additions could be included to design synthetic self-assemblies that can be guided to follow a pathway to preferentially form a specific outcome out of many possible outcomes; the latter being a hallmark of biological and natural self-assembly.

## Materials and Methods

### Experimental details

For our self-assembly experiments, we designed panel and hinge masks using AutoCAD and used them to procure commercial transparency photomasks. We patterned the 2D nets composed of nickel panels and tin-lead solder hinges using photolithography, thin film evaporation, electrodeposition and wet etching. We released the patterned nets from the substrate and heated at ∼200°C in a high boiling point organic solvent. On heating, molten hinge material drives self-assembly of octahedra via surface energy minimization. We designed masks such that the 11 nets were randomly distributed on the wafer to minimize any bias during processing. In order to obtain a statistically significant data set, we processed a total of 60 samples for each of the 11 nets and self-assembled these samples in six batches. Based on these experiments, we observed that several nets self- assembled into both isomers with varied yields and increased propensity for Isomer I formation. We processed a total of 50 samples for each of the two engineered nets to generate the data shown in Fig. 4C. The details of our algorithmic approach to model self-assembly pathways, kinetics, and computation of degrees of freedom of intermediates are given in the supporting information.

## Supporting Information

Figure S1 **Sachse's paper models and corresponding molecular models of cyclohexane.** (A–E) chair conformation: (A) shows the 2D net that generates chair conformation of cyclohexane when folded along the edges *bc, cd, de, ef, fg* and the edges *ab* and *gh* are glued together; and van't Hoff tetrahedron is attached on the dark triangles. The center of each van't Hoff tetrahedron represents carbon atom. (B) and (C) are top views of Sachse's paper model and corresponding ball-stick molecular model, (D) and (E) are side views of Sachse's paper model and corresponding ball-stick molecular model of chair form of cyclohexane; (F–J) boat conformation of cyclohexane: (F) the two nets shown are when folded along the edges and the vertices a, b, c are glued together and van't Hoff tetrahedron is attached on each dark triangle, generate boat form of cyclohexane. (G) and (H) are top views of Sachse's paper model and corresponding ball-stick model and (I) and (J) are side views of Sachse's paper model and corresponding molecular model of boat conformation of cyclohexane.
(TIF)

Figure S2 **The states of configuration space** ***Є*** **for the octahedron self-assembly.** This configuration state is obtained as a result of gluing at vertex connections at both exterior angles 120° and 180°. Configuration space *Є* comprises of 84 states of which states 1 through 11 are initial states, states 83 (Isomer I) and state 84 (Isomer II) are final states, and states 71, 73 and 80 are kinetically trapped states.
(TIF)

Figure S3 Snapshots of a self-assembly movie (Movie S1) showing assembly pathways of net 10 into Isomer II (state 84) proceeding through the intermediate 72, highlighted by the red box.
(TIF)

Figure S4 **Configuration space and dominant intermediates based on shortest path calculations.** (A) and (B) are computed pathways based on greedy algorithms and geodesics for the octahedron self-assembly. As in Fig. 2, the paths in red correspond to states linked by gluing at vertex connections with exterior angle 120° (configuration space *R*) and the edges in green link states obtained by gluing at both types of vertex connections with exterior angles 120° and 180°.
(TIF)

Figure S5 **Engineering self-assembly pathways by manipulating design constraints to enrich formation of Isomer II.** (A) optical image of octahedron net 10 and SEM images of self-assembled isomers I and II; (B) optical image of an engineered net identical to net 10 but one outer panel removed and SEM images of self-assembled isomers I and II. The red triangles represent the open face because of the removed panel; (C) relative yields of isomers I and II formed from the octahedron nets shown in (A) and (B). The scale bar is 300 µm.
(TIF)

Movie S1 Self-assembly of octahedral Isomer II.
(AVI)

Text S1 Gluing algorithm for self-assembly.
(DOCX)

Text S2 Geodesic pathways, dominant intermediates and the kinetics of self-assembly.
(DOCX)

Text S3 Beyond degrees of freedom –quantifying mobility and rigidity of linkages.
(DOCX)

Text S4 Degrees of freedom of Sachse's polyhedral model of cyclohexane.
(DOCX)
