# Supplementary figures and images for: Self-Assembly of Mesoscale Isomers: The Role of Pathways and Degrees of Freedom
Source: PLoS One. 2014 Oct 9;9(10):e108960. doi: 10.1371/journal.pone.0108960 (PMC4191966; doi:10.1371/journal.pone.0108960)

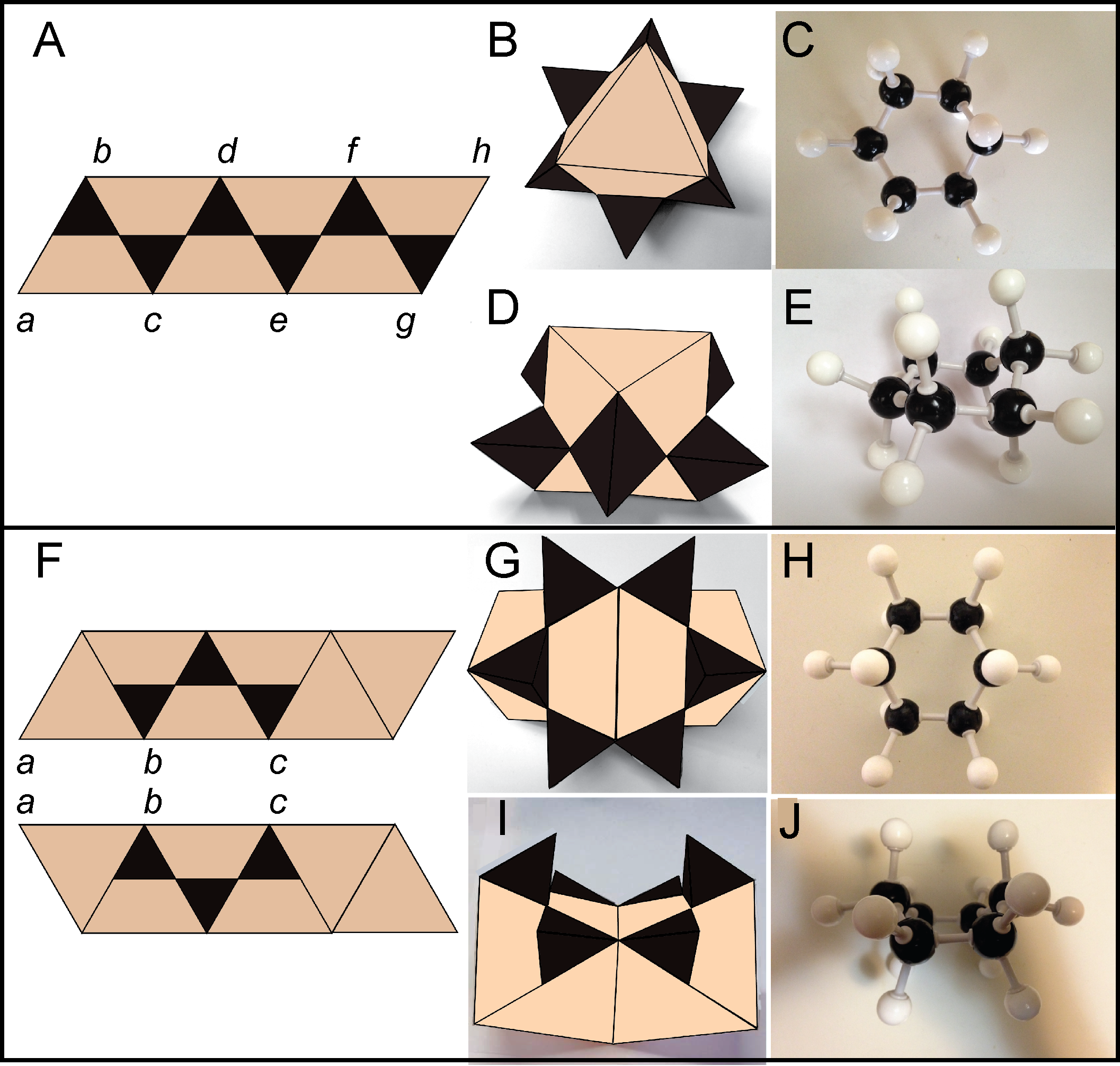

Supplement: Figure S1 — Sachse's paper models and corresponding molecular models of cyclohexane. (A–E) chair conformation: (A) shows the 2D net that generates chair conformation of cyclohexane when folded along the edges bc, cd, de, ef, fg and the edges ab and gh are glued together; and van't Hoff tetrahedron is attached on the dark triangles. The center of each van't Hoff tetrahedron represents carbon atom. (B) and (C) are top views of Sachse's paper model and corresponding ball-stick molecular model, (D) and (E) are side views of Sachse's paper model and corresponding ball-stick molecular model of chair form of cyclohexane; (F–J) boat conformation of cyclohexane: (F) the two nets shown are when folded along the edges and the vertices a, b, c are glued together and van't Hoff tetrahedron is attached on each dark triangle, generate boat form of cyclohexane. (G) and (H) are top views of Sachse's paper model and corresponding ball-stick model and (I) and (J) are side views of Sachse's paper model and corresponding molecular model of boat conformation of cyclohexane. (TIF) [file pone.0108960.s001.tif]

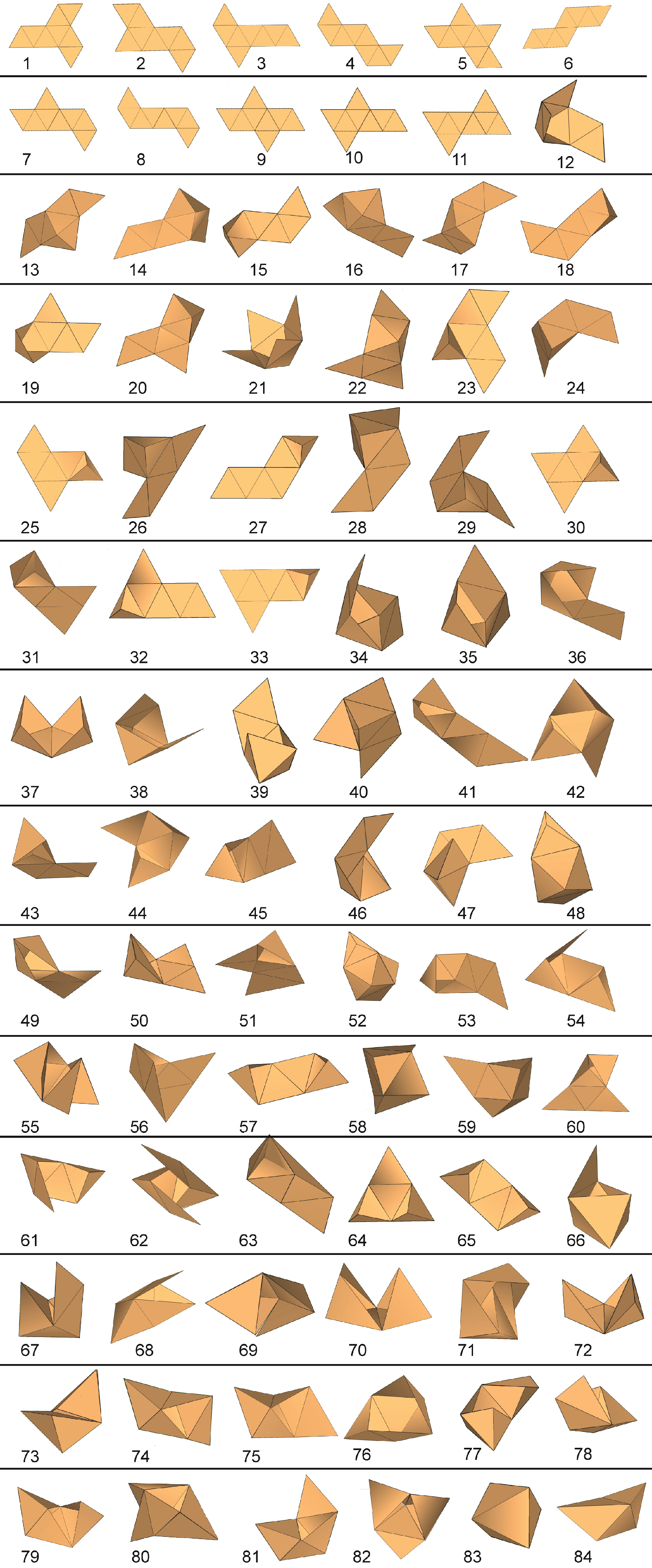

Supplement: Figure S2 — The states of configuration space Є for the octahedron self-assembly. This configuration state is obtained as a result of gluing at vertex connections at both exterior angles 120° and 180°. Configuration space Є comprises of 84 states of which states 1 through 11 are initial states, states 83 (Isomer I) and state 84 (Isomer II) are final states, and states 71, 73 and 80 are kinetically trapped states. (TIF) [file pone.0108960.s002.tif]

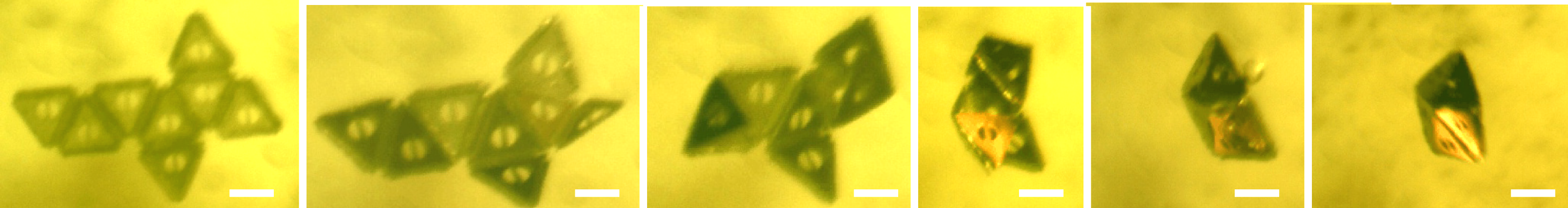

Supplement: Figure S3 — Snapshots of a self-assembly movie (Movie S1) showing assembly pathways of net 10 into Isomer II (state 84) proceeding through the intermediate 72, highlighted by the red box. (TIF) [file pone.0108960.s003.tif]

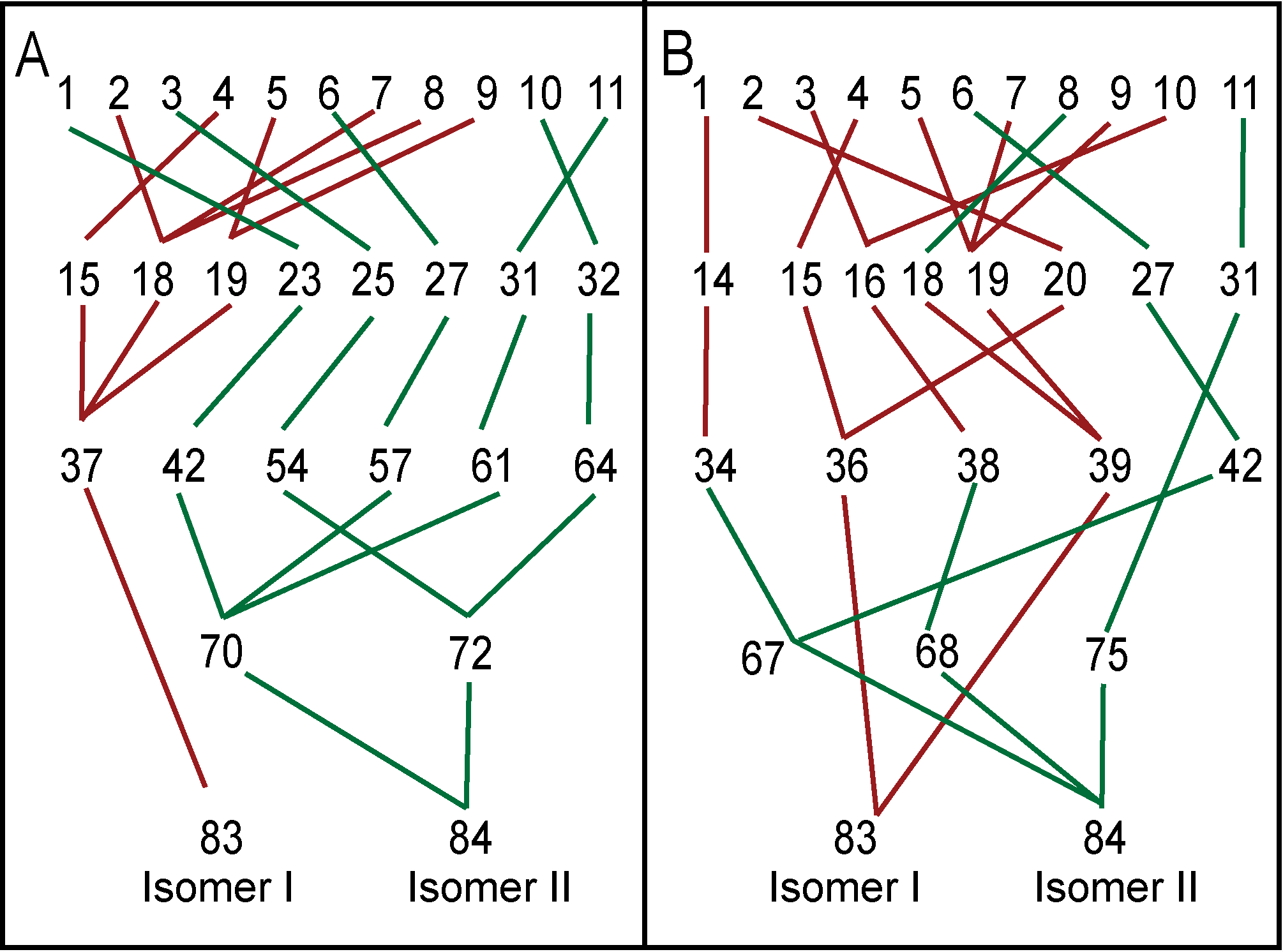

Supplement: Figure S4 — Configuration space and dominant intermediates based on shortest path calculations. (A) and (B) are computed pathways based on greedy algorithms and geodesics for the octahedron self-assembly. As in Fig. 2, the paths in red correspond to states linked by gluing at vertex connections with exterior angle 120° (configuration space R) and the edges in green link states obtained by gluing at both types of vertex connections with exterior angles 120° and 180°. (TIF) [file pone.0108960.s004.tif]

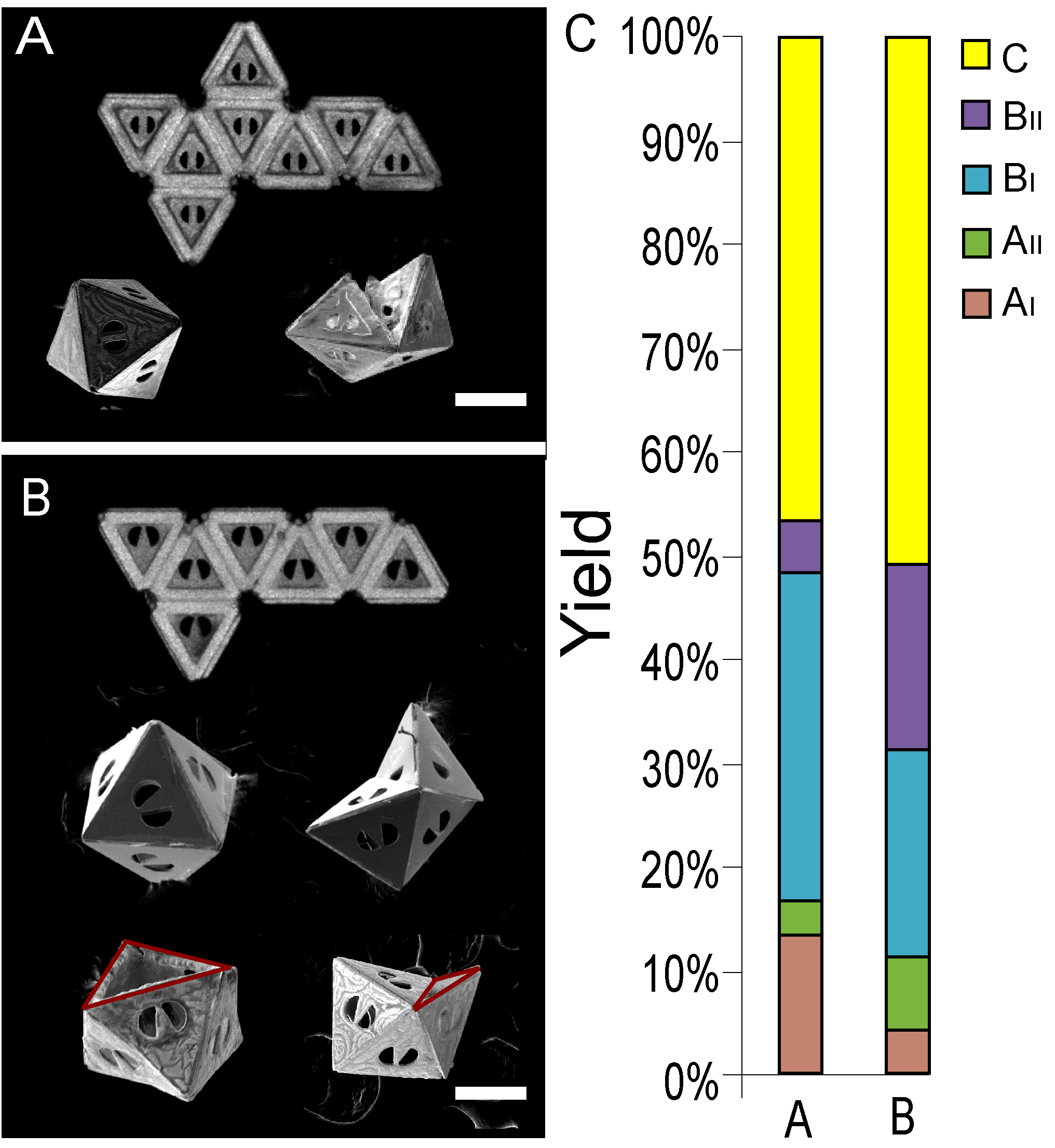

Supplement: Figure S5 — Engineering self-assembly pathways by manipulating design constraints to enrich formation of Isomer II. (A) optical image of octahedron net 10 and SEM images of self-assembled isomers I and II; (B) optical image of an engineered net identical to net 10 but one outer panel removed and SEM images of self-assembled isomers I and II. The red triangles represent the open face because of the removed panel; (C) relative yields of isomers I and II formed from the octahedron nets shown in (A) and (B). The scale bar is 300 µm. (TIF) [file pone.0108960.s005.tif]
