# Supplementary material for: Self-Assembly of Mesoscale Isomers: The Role of Pathways and Degrees of Freedom
Source: PLoS One. 2014 Oct 9;9(10):e108960. doi: 10.1371/journal.pone.0108960 (PMC4191966; doi:10.1371/journal.pone.0108960)
Supplement: Text S1 — Gluing algorithm for self-assembly. (DOCX) [file pone.0108960.s007.docx]

**1. Gluing algorithm for self-assembly**

In order to construct the configuration space, we begin at a net and proceed with an algorithm called gluing at vertex connections with exterior angle 120^0^ until a final folded state is reached. An analogy could be made between gluing of edges and the formation of a stable bond during self-assembly of octahedra. Briefly the algorithm is as follows:

1. Begin with a net *S­_0_*.

2. Choose any vertex connection *v_0_* of *S_0_*, and glue the emanating edges. These may only be glued by rotating rigidly the dihedral angle about self-assembly hinges that meet at *v_0._* Let *S_1_* be the new set of faces, edges, and vertices formed by this gluing.

3. Continue recursively by defining *S_k+1_* as the gluing of a vertex connection *v_k_* in *S_k_*.

4. Terminate the process when there are no available vertex connections, or gluing is impossible.

For a process terminating after *n* *gluings*, the resulting set of states (*S_0_*, . . . , *S_n_*) produced is called a pathway of *S­_0_*; e.g. the set {1, 12, 34, 66, 83} shown in Figure 2 is a self-assembling pathway of net 1, where state 1 is the starting net (*S_0_*), state 83 is the final state (*S_n_*) and states 12, 34 and 66 are intermediates. When panels at vertex connection 120^0^ are glued, they always meet at the dihedral angle corresponding to an octahedron (83). As a consequence, all pathways terminate at a unique state — a perfectly folded convex octahedron (Isomer I). The complete set of states that results from all possible nets of the octahedron by gluing at vertex connections with 120^0^ angle is the configuration space *C* of the octahedron comprising of 30 states. These 30 states can be divided into five transition states of intermediates where the states {1, 2, 3, 4, 5, 6, 7, 8, 9, 10, 11} represent initial states, the sets {12, 13, 14, 15, 16, 17, 18, 19, 20, 21, 22}, {34, 35, 36, 37, 38, 39} and {66} represent intermediate transition states and {83} is the final state. When gluing algorithm is followed for both types of vertex connections at 120^0^ and 180^0^ angles, the complete set of states we obtain is the configurations space *Є,* comprising of 84 states*.*
