# Supplementary material for: Self-Assembly of Mesoscale Isomers: The Role of Pathways and Degrees of Freedom
Source: PLoS One. 2014 Oct 9;9(10):e108960. doi: 10.1371/journal.pone.0108960 (PMC4191966; doi:10.1371/journal.pone.0108960)
Supplement: Text S2 — Geodesic pathways, dominant intermediates and the kinetics of self-assembly. (DOCX) [file pone.0108960.s008.docx]

**2. Geodesic pathways, dominant intermediates and the kinetics of self-assembly**

The intermediate states represented by S_0_, S_1_, S_2_, S_3_ and S_4_ have 7, 5, 3, 1 and 0 degrees of freedom respectively. Thus, the number of degrees of freedom allows us to clearly quantify the increase of rigidity as self-assembly proceeds. However, it does not allow us to distinguish between intermediates on the same tier. We do not expect all of these intermediates to be equally important in self-assembly. In order to gain further insight into the intermediate states that dominate the self-assembly pathways, we adapt ideas from our previous work and analyze the paths from the nets on tier S_0_ to the terminal states on tier S_4_ that minimize suitable cost functions; and the cost function we use is a simplified version of the distance function used earlier [1]. We distinguish between geodesic paths (those that minimize the total distance) and greedy paths (those that minimize the distance at each step). According to the greedy path minimization as shown in Figure S4 (a), nets 2, 4, 5, 7, 8 and 9 terminate at state 83 and notably all of these paths focus through intermediate 37. The geodesics (Figure S4 (b)) that minimize the global paths between the initial net and state 83 proceed through intermediates 36, 39 and do not focus as sharply through particular intermediates when compared with the greedy path computation. Similarly for formation of Isomer II, greedy paths focus sharply through intermediates 70 and 72.

Thus, it is clear that the greedy paths are more reliable measures to find most prevalent intermediates than geodesics. In addition to thinning down the set of intermediates, the calculation of greedy paths also suggests that nets 1, 3, 6, 10 and 11 are more likely to form boats than nets 2, 5, 7, and 8. This trend is well reflected in experiment, as shown in Figures 3 and S3. Other criterion to narrow down the set of dominant intermediates based on degrees of freedom and kinetics of assembly are discussed in SI 1.3.

The configuration space can also be used to model the kinetics of assembly. We may use a stochastic (Markov chain) model on *Є*, or we may model kinetics of self-assembly with rate equations. If we assume that a state $j$ transforms to a state $k$at constant rate *a_i,,k_* , then a population of states *C(t) =( c_1_(t),…c_n_(t))* evolves according to the linear rate :

;  *(1)*

With these equations, we can rank states by a time average

**** (2)

We term the coordinate of the prevalence of state at time. The prevalence provides a kinetic measure of important states. There are as many rate constants as there are edges in the configuration space, and it is difficult to estimate these rates directly from data. Instead, we numerically computed the solution to *(1)* and *(2)* for many different rate constants of the form, for several different positive functions$f$. Remarkably, we find that for all these choices, the most prevalent intermediates are well correlated with the intermediates given by the shortest paths.

**References:**

1. Pandey S, Ewing M, Kunas A, Nguyen N,Gracias DH et al. (2011) Algorithmic design of self-folding polyhedra. Proc Natl Acad Sci USA 108: 19885-19890.
