# Supplementary material for: Self-Assembly of Mesoscale Isomers: The Role of Pathways and Degrees of Freedom
Source: PLoS One. 2014 Oct 9;9(10):e108960. doi: 10.1371/journal.pone.0108960 (PMC4191966; doi:10.1371/journal.pone.0108960)
Supplement: Text S3 — Beyond degrees of freedom –quantifying mobility and rigidity of linkages. (DOCX) [file pone.0108960.s009.docx]

**3. Beyond degrees of freedom—quantifying mobility and rigidity of linkages**

The number of degrees of freedom of a polyhedral linkage is the difference between the number of coordinates required to prescribe the linkage completely and the number of independent constraint equation. The linkage is rigid if it has no internal degrees of freedom (that it, it has precisely six degrees of freedom corresponding to three coordinates for the center of mass and three coordinates to describe orientation). As noted in Figure 2, the process of self-assembly corresponds to an increase in rigidity – at each step of self-assembly, edges are glued. Thus, more constraint equations are added to the system. The final states (the boat and octahedron) are rigid.

As shown in Figure 2, the number of degrees of freedom does not allow us to distinguish between intermediates on the same tier, in order to focus on the most likely or most stable intermediate in self-folding. Finding a more refined measure of rigidity (or mobility) is an interesting question that has not been explicitly treated in the mathematical literature on polyhedra. For example, while all Connelly polyhedra have one degree of freedom, from a practical standpoint it is important to choose edge lengths so that the polyhedron flexes as much as possible [2]. Such a notion of mobility involves going beyond an integer that measures the difference between equations and unknowns, to a careful investigation of how much a linkage can be deformed. The set of admissible deformations of the linkage is the space of solutions to the constraint equations. Such solution spaces are often of intrinsic mathematical interest [3], [4]. In our work we are confronted with the task of assigning a theoretically sound, easily computable measure of deformability to this solution space. Perhaps the most natural measure of deformability is the k-dimensional volume of the solutions space, where k denotes the number of degrees of freedom of the linkage. For example, the possible configurations of each net on tier 0 in Figure 2 constitute a hypersurface of dimension 7. Thus, to measure which of these nets is most easily deformed, it is necessary to measure the 7-dimensional volume of all possible configurations obtained from the net by folding it at each internal hinge, without gluing edges or allowing self-intersections. However, the computation of the k-dimensional volume of the solution space for each intermediate is computationally intractable. We chose instead to test the linearization (i.e. Jacobian matrix) of the constraint equations at certain experimentally observed `canonical’ configurations to see if this serves as a measure of “mobility” of intermediates. For example, in order to test rigidity of intermediates we computed the mobility of four intermediates-34, 36, 37 and 64. Since 36 and 37 form Isomer I, this rigidity was measured using canonical octahedral configuration. From these calculations, we find that intermediate 34 in the canonical octahedron configuration is the most rigid of the intermediates tested. Intermediate 34 in the canonical boat configuration has about the same rigidity as intermediates 36 and 37. Finally, intermediate 64 is the least rigid of the intermediates tested and this least rigid intermediate forms Isomer II. These calculations suggest that intermediates with higher mobility prefer pathways leading to formation of Isomer I.

**References:**

1. Demaine E, O’Rourke J (2007) Geometric Folding Algorithms: Linkages, Origami, Polyhedra, Cambridge University Press, New York, 299-338.
2. Connelly R (1977) A counterexample to the rigidity conjecture for polyhedra. Publ Math IHES 47: 333-338.
3. Thurston, WP (1998) Classical Quant Grav 15(9): 2545.
