# Supplementary material for: Self-Assembly of Mesoscale Isomers: The Role of Pathways and Degrees of Freedom
Source: PLoS One. 2014 Oct 9;9(10):e108960. doi: 10.1371/journal.pone.0108960 (PMC4191966; doi:10.1371/journal.pone.0108960)
Supplement: Text S4 — Degrees of freedom of Sachse's polyhedral model of cyclohexane. (DOCX) [file pone.0108960.s010.docx]

**4. Degrees of freedom of Sachse’s polyhedral model of cyclohexane**

Considering eclipsing and angle strains, we heuristically defined an idealized constraint model by imposing geometric constraints on connectivity between two carbon atoms (bond length) and angular arrangement of bonds in space (angle strain). Each configuration can be represented by locating the centers of carbon atoms in 3D space and representing them as vertices of the graph$v_{1,} v_{2, \ldots}v_{6,}$; $v_{k}\in R^{3};k=1,2,..,6$. For ease of exposition, we use the notation$v_{0}=v_{6}.$ Any two atoms are bonded with a fixed bond length of$l$. Since we can re-scale our coordinate system, we assume $l=1$ and the connectivity in cyclohexane molecule is such that $v_{k}$ is bonded to $v_{k-1}$ for$k=1,2,3..,6$. Upon imposing constraints over the connectivity, we get a set of six constraint equations corresponding to 6 C-C bonds, which can be written as $0=\varphi_{len}^{k}\left( v_{k-1},v_{k} \right)=|v_{k}$-$v_{k-1}|-1;k=1,2,3..,6.$ Since the carbon atoms have the lowest energy in tetrahedral geometry, we fix the angle of each set of three adjacent carbon atoms to be at the tetrahedral geometry and imposing this constraint on angular arrangement of atoms, we get another set of 6 constraint equations represented by the dot product of two bonds, $0=Ф_{ang}^{k}\left( v_{k-2},v_{k-1}, v_{k} \right)=\left( v_{k}-v_{k-1} \right).\left( v_{k-2}-v_{k-1} \right)+1/3;k=1,2,3,..6.$ (Since any configuration also has infinitely many equivalent configurations given by rotations and translations, we also fix the first three atoms $v_{1,} v_{2,}v_{3,}$ at positions $c_{1,} c_{2,}c_{3}$).We then verified that both the boat and chair configurations solved the constraints equations exactly. This fact is well-known (though it was obscured by Sachse’s cumbersome analysis (see Figure S1). A more surprising finding involves the (ideal) rigidity of these configurations. We find that the chair is rigid (has zero degree of freedom), while the boat has one-degree of freedom. These degrees of freedom were computed by computing the rank of the Jacobian matrix of the constraint equations evaluated at the chair and boat configurations.
